# Effects of epiphytic and exogenous lactic acid bacteria on fermentation quality and microbial community compositions of paper mulberry silage

**DOI:** 10.3389/fmicb.2022.973500

**Published:** 2022-08-25

**Authors:** Qiming Cheng, Maoya Li, Xueying Fan, Yulian Chen, Hong Sun, Yixiao Xie, Yulong Zheng, Chao Chen, Ping Li

**Affiliations:** ^1^College of Animal Science, Guizhou University, Guiyang, China; ^2^Sichuan Academy of Grassland Sciences, Chengdu, China; ^3^Key Laboratory of Animal Genetics, Breeding and Reproduction in the Plateau Mountainous Region, Ministry of Education, Guizhou University, Guiyang, China

**Keywords:** lactic acid bacteria, paper mulberry, silage, fermentation quality, microbial community

## Abstract

This study aimed to isolate, characterize, and identify lactic acid bacteria (LAB) strains from various sources and evaluate their effects on the nutritional quality, fermentation characteristics, and microbial compositions of paper mulberry (PM) after 60 days of ensiling. Forty-nine LAB strains were isolated from *Phalaris arundinacea* silage, pickle, and fresh PM leaves; three of these strains (*Lactiplantibacillus plantarum*, YC1; *Levilactobacillus brevis*, PC3; and *Lactiplantibacillus plantarum*, BP17) and one commercial inoculant Gaofuji (GFJ) were subsequently used. Compared with other treatments, PC3 and BP17 increased (*P* < 0.05) the LAB count and crude protein content and decreased (*P* < 0.05) the molds and coliform bacteria counts, pH, and ammonia-N content of PM silages. BP17 and PC3 increased the relative *Lactiplantibacillus* abundance and decreased that of *Lelliottia* and *Cladosporium*, improving PM silage quality. Therefore, PC3 and BP17 can improve the fermentation quality of PM silage and could be used as silage starter cultures.

## Introduction

With the rapid development of animal husbandry in China, traditional feeds such as fodder crops, grasses, and grain are insufficient to meet the demand for livestock. The development and utilization of new feed resources has proven to be a viable solution to the feed crisis. Recently, woody forage processing and feeding technology have been studied in China (Si et al., 2018; Zhang et al., 2019a). Paper mulberry (PM; *Broussonetia papyrifera* L.), a typical woody forage, is fast growing; rich in crude protein (211.50–245.92 g/kg of DM), amino acids and flavonoids; and widely distributed in Asia (Pang et al., 2014; Cheng et al., 2021a; Wang et al., 2021a). The PM is widelyx distributed, with annual production of about 2.25 × 10^8^ t in temperate and tropical zones of China (Dong et al., 2020). As one of the country’s top 10 targeted poverty alleviation initiatives, China has planted more than 300,000 hectares of PM for use as an unconventional animal feed (Hao et al., 2021). There have been many studies on the nutritional value of PM and the effects of feeding PM on livestock. For example, some studies found that adding an appropriate amount of PM silage to the diets of cattle and goats could improve feed efficiency, growth performance, meat quality, and immune and antioxidant function (Hao et al., 2020; Hua et al., 2020; Tao et al., 2020; Tian et al., 2020). Another study revealed the use of PM as a new type of animal feed that could be a candidate protein feed resource in response to the feed crisis (Guo et al., 2021).

PM is harvested during the rainy season, resulting in a high moisture content, and ensiling has been indicated to be the best way to preserve PM (Zhang et al., 2019a). In the process of ensiling, epiphytic lactic acid bacteria (LAB) ferment soluble carbohydrates in fresh forage into organic acids, mainly lactic acid, thereby reducing pH and inhibiting harmful microorganisms (Dong et al., 2019). As a result, ensiling is a microbial-driven process, in which LAB play a key role. Our previous studies have shown that without treatment, PM is difficult to ensile well due to the considerably high buffering capacity and the low epiphytic LAB count [< 10^5^ cfu/g of fresh matter (FM)] of fresh forage (Cheng et al., 2021a). Exogenous LABs are frequently used to speed up the process of ensiling, prevent the growth of harmful microorganisms, and improve the silage quality of PM (Du et al., 2021; Wang et al., 2021b). Because the adaptability, establishment, and development of LAB in forages during ensiling is unknown, the conditions used do not always result in successful regulation of silage fermentation with LAB inoculants (Kobayashi et al., 2010; Tohno et al., 2012). Wang et al. (2021b) found that adding LAB [*Lactiplantibacillus plantarum* (*L. plantarum*) or *Lacticaseibacillus casei*] isolated from *Leymus chinensis* silage did not significantly improve the fermentation quality of PM silage, which was mainly reflected in the high pH (> 6) value and ammonia nitrogen (NH_3_–N) content (>16% TN), indicating that these LAB strains were not the best choice for PM ensiling. Previous studies have shown that adding LAB (isolated from fresh PM leaves) can improve the fermentation quality of PM silage (Cheng et al., 2021a). According to previous research, the best isolates for boosting fermentation quality may originate from that specific forage (Zhang et al., 2015; Wang et al., 2018a). Therefore, it is necessary to explore LAB strains that are adaptable and can play a role in PM silage. However, to our knowledge, few studies have focused on the effects of epiphytic LAB isolated from various forage sources on the fermentation quality of PM silage.

It is widely established that bacterial community structure and abundance are key factors that affect the fermentation quality of silage. Previously published studies indicated that a high abundance of harmful bacteria (such as *Enterobacter* or *Clostridium*) and a low abundance of beneficial bacteria (such as *Lactobacillus*) in silage were the main challenges for proper PM ensiling (Cheng et al., 2021a; Du et al., 2021). However, the structure and abundance of fungal community members (such as *Saccharomyces*, *Cladosporium* or *Issatchenkia*) during the ensiling process also affected the fermentation quality of silage (Li et al., 2021). Therefore, the composition and shift in bacteria and fungi may affect the fermentation quality of PM silage. In recent years, 16S rRNA and ITS sequencing has changed our understanding of bacterial and fungal communities in fresh and ensiled forages, including alfalfa (Bai et al., 2020), sugarcane (Wang et al., 2020), and timothy (Li et al., 2021). To the best of our knowledge, there have been no reports on the diversity of the fungal community in fresh and ensiled PM. Hence, the objective of the present study was to compare the effects of specific LAB, including epiphytic, exogenous, and commercial LAB inoculants, on the nutritional quality, fermentation characteristics, and bacterial and fungal community compositions of PM silage. Our hypothesis was that LAB strains isolated from different sources could functionally improve the silage quality of PM.

## Materials and methods

### Isolation, screening, characterization, and identification of lactic acid bacteria isolated from different sources

A total of 49 LAB strains were isolated from *Phalaris arundinacea* (*P. arundinacea*) silage, pickle, and fresh PM leaves according to the method of Cai et al. (1998). Ten grams of sample from each material was put into a sterile glass bottle and blended with 90 mL of sterile water. Serial dilutions were used for the isolation of LAB using de Man, Rugose, Sharpe (MRS) agar (GCM188, Land Bridge Technology Co., Ltd., Beijing, China). The morphological characteristics, growth ability, and acid production capacity of all identified strains were measured on MRS agar medium. The physiological and biochemical features of three LAB strains (*L. plantarum* isolated from *P. arundinacea* silage, YC1; *L. plantarum* isolated from pickle, PC3; and *Levilactobacillus brevis* isolated from fresh PM leaves, BP17) with rapid growth ability and high acid production ability were investigated. In addition, the tested strains were genetically identified by 16S rRNA gene sequencing (Sangon Biotech (Shanghai) Co., Ltd., Shanghai, China) and preserved in China General Microbiological Culture Collection Center under accession numbers CGMCC NO. 17813 (YC1), CGMCC No. 17726 (PC3) and CGMCC No. 17814 (BP17), respectively.

### Silage preparation

This study was conducted at the experimental base (Chengdu) of Sichuan Academy of Grassland Science (103°22′E, 33°33′N). Whole PM leaves were harvested as ensiling material on July 1, 2020 and chopped to a length of 2–3 cm. Silages were prepared on a small-scale system by packing 500 g of chopped PM leaves without or with LAB inoculants into polyethylene bags (25 cm × 30 cm) and then vacuum packed using a vacuum packing machine (SJ-400, Shanghai Precision Machinery Manufacturing Co., Ltd.). The treatments were as follows: (1) CK, control without additives, treated with 5 mL kg^–1^ FM 0.9% physiological saline; (2) YC1, applied at 1.0 × 10^6^ cfu g^–1^ of FM; (3) PC3, applied at 1.0 × 10^6^ cfu g^–1^ of FM; (4) BP17, applied at 1.0 × 10^6^ cfu g^–1^ of FM (Cheng et al., 2021a); (5) Gaofuji (GFJ), a combination of *L. plantarum* and *Lentilactobacillus buchneri*, produced by Sichuan Gaofuji Biotechnology Co., Ltd., applied at 1.0 × 10^6^ cfu g^–1^ of FM. Three polyethylene bags of silage with the same treatment were sampled for analysis after 60 d of ensiling in a dark room at room temperature (20–25°C). Samples from fresh and ensiled PM leaves were subjected to analyses of chemical composition, fermentation characteristics, microbial population, and bacterial and fungal community compositions.

### Analysis of microbial population, chemical composition, and fermentation quality

The microbial populations on fresh material and silage samples were determined according to the method of Cai et al. (1999). Ten grams of each sample was suspended in 90 mL of sterilized water and serially diluted from 10^–1^ to 10^–5^. The number of LAB was measured by plate counting on MRS agar (GCM188, Land Bridge Technology Co., Ltd., Beijing, China) and kept in an anaerobic incubator for 48 h at 37°C. Molds and yeasts were incubated in a general incubator on malt extract agar (CM173, Land Bridge Technology Co., Ltd., Beijing, China) for 48 h at 30°C, and yeasts were distinguished from molds by colony appearance and cell morphology. Aerobic bacteria were counted on nutrient agar (Nissui) and incubated for 48 h at 30°C under aerobic conditions. Coliform bacteria were incubated on blue light broth agar (Nissur Ltd., Tokyo, Japan) for 48 h at 30°C. The microbial counts were expressed as log cfu/g of FM.

Oven drying at 65°C to constant weight was used to determine the dry matter (DM) content of fresh material and silage samples. All dried samples were milled to pass through a 0.20 mm screen for determination of the chemical compositions. The total nitrogen (TN) content was determined using a Kjeldahl nitrogen analyzer (Kjeltec 8400, FOSS, Sweden), and crude protein (CP) was calculated by multiplying TN by 6.25 (AOAC, 1990). The neutral detergent fiber (NDF) and acid detergent fiber (ADF) levels were analyzed with a modified procedure using an ANKOM 2000 Fiber Analyzer (ANKOM Technology Corp., Fairport, NY, United States) (Van Soest et al., 1991). The water-soluble carbohydrate (WSC) was determined using the method of Mcdonald et al. (1991). The *in vitro* ruminal DM digestibility (IVDMD) of all silage samples was determined according to the method of Goto and Minson (1977) using the two-stage fermentation technique.

A silage sample of 10 g was mixed with 90 mL ultrapure water for 3 min in a stomacher blender. The pH value of the silage extract was determined by a pH meter (3-Star 310P-02, Thermo Electron, Boston, United States). The ammonia nitrogen (NH_3_–N) content was determined in the silages by the phenol-hypochlorite procedure (Kleinschmit et al., 2005). A filtrate of approximately 10 mL was subjected to centrifugation (12,000 × g, 10 min, 4°C), and the lactic acid (LA), acetic acid (AA), propionic acid (PA), and butyric acid (BA) contents in the supernatant were analyzed using high-performance liquid chromatography (HPLC) (LC-20A; Shimadzu, Tokyo, Japan) with a UV detector (210 nm) and a column (e2695, Waters Co., Ltd.) (Cheng et al., 2021b).

### Sequencing-based microbial analyses

Microbial DNA was extracted from the fresh PM and silage according to the method described in Li et al. (2021). In brief, a Power Soil DNA Isolation Kit (MO BIO Laboratories) was used to extract microbial DNA following the manufacturer’s instructions. All microbial DNA samples were immediately sent to Novogene Company (Beijing, China) for PCR amplification and bioinformatic analysis. The primers 341F (5′-CCTAYGGGRBGCASCAG-3′) and 806R (5′-GGACTACNNGGGTATCTAAT-3′) were chosen to amplify the V3-V4 region of the 16S rRNA gene. The primers ITS1F (5′– CTTGGTCATTTAGAGGAAGTAA–3′) and ITS2-2043R (5′-GCTGCGTTCTTCATCGATGC-3′) were used to amplify the ITS gene (Li et al., 2021). Novogene (Beijing, China) completed library construction and Illumina S5 sequencing according to the manufacturer’s instructions. The data were analyzed using the Novogene Magic Cloud Platform.^1^

### Statistical analyses

Data on the microbial population, chemical composition and fermentation quality of fresh and ensiled paper mulberry were analyzed using one-way analysis of variance (ANOVA) to evaluate the effects of LAB inoculants. The differences between means were assessed using Duncan’s multiple range method. The effect was considered significant when *P* < 0.05. The analyses were conducted using IBM SPSS Statistics 26.0 (SPSS, Inc., Chicago, IL).

## Results and discussion

### Characteristics and identification of selected lactic acid bacteria strains

More than 200 strains were isolated from different sources, and Gram staining, colony morphology and catalase activity tests identified 49 of them as LAB strains. Three strains, YC1, PC3, and BP17, were selected for further study according to their growth ability and acid production ability. The morphological, physiological, and biochemical properties of the three LAB strains used in our study are shown in Table 1. Previous studies reported that 16S rDNA sequencing was a good method for identifying microorganisms by genus and species (Cai et al., 1998; Wang et al., 2019a). In our study, 16S rDNA sequence analysis was used to identify these three strains. Among them, YC1 isolated from *P. arundinacea* silage and BP17 isolated from fresh PM leaves were *L. plantarum*, and PC3 isolated from pickle was *Levilactobacillus brevis*. The YC1 and BP17 strains were rod shaped, homofermentative, Gram positive, catalase negative, and glucose negative. This result is consistent with the study of Guo et al. (2020), who reported that F1 and F50 isolated from feces were *L. plantarum* and could be used to improve the silage quality of alfalfa. The PC3 strain was rod shaped, heterofermentative, Gram positive, catalase negative, and glucose positive. Previous studies revealed that *Levilactobacillus brevis* isolated from pickle had antifungal, antioxidant, and probiotic properties, and it has been successfully used in silage (Grant et al., 1994; Arasu et al., 2015). The YC1 strain could grow normally at temperatures from 5 to 35°C, while the PC3 and BP17 strains could grow normally at 15–40°C. This indicates that the YC1 strain is more resistant to low temperature than the PC3 and BP17 strains. The raw material from which YC1 was isolated (*P. arundinacea* silage) came from the Qinghai-Tibetan Plateau, while the raw materials from which PC3 (pickle) and BP17 (fresh leaves of PM) were isolated came from Chengdu; therefore, this difference in optimal growth temperature could have arisen due to long-term evolution and natural selection on the different environmental temperatures (You et al., 2021). This unique characteristic of the YC1 strain suggests that it may be used as a low-temperature-tolerant LAB inoculant, consistent with the results of Chen et al. (2020a), who reported that *L. plantarum* isolated from naturally fermented silage on the Qinghai Tibetan Plateau could grow normally at 5–30°C. The YC1 and BP17 strains could grow normally at pH values ranging from 3.0 to 8.0, while the PC3 strain could grow normally at pH 4–8, and all isolated strains could grow normally with NaCl concentrations of 3% (w/v) and 6.8% (w/v). These findings indicated that the tolerance of PC3 to an acidic environment was weaker than that of YC1 and BP17. This is because the PC3 strain is *Levilactobacillus brevis*, a heterofermentative LAB that plays a role in the early period of ensiling and is less resistant to the acidic environment than *L. plantarum* (Zheng et al., 2017; Amaral et al., 2020). This finding is consistent with the results of Wang et al. (2018a), who reported that *L. plantarum* was highly tolerant to low pH. The ability to reduce the pH of the medium has long been one of the main principles for selecting LAB as inoculants (Liu et al., 2014). In this study, the strains were inoculated in MRS medium and cultured at 30°C for 24–72 h. The pH ranges of the MRS medium from YC1, PC3, and BP17 were 4.00–4.96, 3.83–3.96, and 3.85–3.93, respectively. All of the strains could produce acid from galactose, lactose, sucrose, maltose, cellobiose, and inulin, which indicated that the strains isolated in our study had a wide variety of fermentation substrates. The selected strains, YC1, PC3, and BP17, demonstrated a wide range of temperature, pH, and salt tolerances, as well as a high acid production and a wide variety of fermentation substrates. These unique properties of these three selected strains offer the potential for practical applications as inoculants.

**TABLE 1:** Morphological, physiological, and biochemical properties of lactic acid bacteria isolates.

Items	Strains		
	
	YC1	PC3	BP17
Sources	*Phalaris arundinacea* silage	Pickle	Fresh leaves of PM
Species	*Lactiplantibacillus plantarum*	*Levilactobacillus brevis*	*Lactiplantibacillus plantarum*
Shape	Rod	Rod	Rod
Fermentation type	Ho	He	Ho
Gram strain	+	+	+
Catalase activity	-	-	-
Gas for glucose	-	+	-
Growth at temperature (°C)			
5	+	-	-
10	+	w	w
15	+	+	+
20	+	+	+
25	+	+	+
30	+	+	+
35	+	+	+
40	w	+	+
45	w	w	+
Growth at pH			
3	+	-	+
3.5	+	w	+
4	+	+	+
4.5	+	+	+
5	+	+	+
6	+	+	+
7	+	+	+
8	+	+	+
Growth in NaCl, % (w/v)			
3	+	+	+
6.8	+	+	+
Acid production (pH value)			
24 h	4.96	3.83	3.85
48 h	4.00	3.96	3.92
72 h	4.03	3.94	3.93
Carbohydrate fermentation			
Galactose	+	+	+
Lactose	+	+	+
Sucrose	+	+	+
Maltose	+	+	+
Cellobiose	+	+	+
Inulin	+	+	+

YC1, Lactiplantibacillus plantarum; PC3, Levilactobacillus brevis; BP17, Lactiplantibacillus plantarum; PM, paper mulberry; He, heterofermentative; Ho, homofermentative; w, weak; +, positive; -, negative.

### Microbial counts and chemical compositions of fresh and ensiled paper mulberry

The microbial populations of fresh and ensiled PM are presented in Table 2. A low count of LAB (10^2.75^ cfu/g of FM) was detected in the fresh PM, and high counts of undesirable microorganisms, including molds, yeast, aerobic bacteria, and coliform bacteria, were detected (10^3.89^–10^6.19^ cfu/g FM). The number of epiphytic LAB on the fresh PM (10^2.48^ cfu/g of FM) in our study was similar to the result reported by Cheng et al. (2021a). In our study, the low count of epiphytic LAB (< 10^5^ cfu/g of FM) and high count of harmful microorganisms on fresh PM made it difficult to ensile well, and additional LAB must be added to improve silage quality. As expected, as fermentation progressed, the counts of LAB increased (> 10^5^ cfu/g of FM), whereas the counts of undesirable microorganisms decreased (*P* < 0.05). Similarly, Cheng et al. (2021b) reported that the number of helpful microorganisms increased, and the number of harmful microorganisms decreased after ensiling. Moreover, compared with other treatments, the PC3 and BP17 treatments had a higher (*P* < 0.05) number of LAB and lower (*P* < 0.05) numbers of molds and coliform bacteria.

**TABLE 2:** The microbial population by plate culture of fresh and ensiled paper mulberry.

Samples	LAB	Molds	Yeasts	Aerobic bacteria	Coliform bacteria
	
	(log cfu/g of FM)				
Fresh					
	2.75c	4.81a	3.89a	6.19a	5.23a
Silage					
YC1	5.83b	4.03b	2.34b	3.82b	3.62b
PC3	8.92a	1.72d	2.62b	3.79b	2.03c
BP17	8.63a	1.27d	2.49b	3.68b	2.21c
CK	5.88b	3.05c	2.46b	3.81b	3.77b
GFJ	5.43b	3.18c	2.54b	3.77b	3.84b
SEM	0.91	0.42	0.29	0.61	0.22
*P*-value	0.005	0.032	0.046	0.033	0.044

^a–d^Means in the same column with different letters differ significantly from each other (P < 0.05).

LAB, lactic acid bacteria; FM, fresh matter; cfu, colony forming units; YC1, Lactiplantibacillus plantarum; PC3, Levilactobacillus brevis; BP17, Lactiplantibacillus plantarum; CK, control without additives, applied at 5 mL kg^–1^ FM 0.9% physiological saline; GFJ, Gaofuji, a commercial inoculant containing Lactiplantibacillus plantarum and Lentilactobacillus buchneri; SEM, standard error of means.

The chemical compositions of fresh and ensiled PM are displayed in Table 3. Studies have found that the presence of a sufficient WSC content (6–8% DM) in the fresh material is essential to ensure the fermentation quality of silage (Wang et al., 2018a). In our study, the WSC concentration (7.58% DM) of fresh PM was enough to promote LAB fermentation. After 60 d of ensiling, the YC1, PC3, and BP17 treatment silages consistently showed lower (*P* < 0.05) WSC contents than the CK and GFJ treatment silages. This may be due to the conversion of WSC content into acid, ethanol, and carbon dioxide by LAB during fermentation (Muck et al., 2018). All LAB treatments showed a greater (*P* < 0.05) CP concentration than the CK treatment, which could be due to LAB limiting clostridia growth and proteolysis (Arriola et al., 2011). Among the LAB treatments, PC3 and BP17 showed the best inhibitory effect, mainly due to their significantly higher CP content. The NDF and ADF levels of fresh PM were 30.39% DM and 18.30% DM, respectively, which were lower than the results (50.28% DM and 34.89% DM, respectively) reported in Hao et al. (2021). This is because previous studies focused on the whole PM plant, while this study focused on the PM leaves. The PC3 and BP17 treatments resulted in significantly lower (*P* < 0.05) NDF and ADF levels than the GFJ and YCI treatments, which indicated that PC3 and BP17 were more capable of degrading fiber than other LAB additives. Feeding well-fermented silage helps to improve animal performance because of the high level of LA and the low level of AA and ammonia nitrogen in the silage (Wang et al., 2019b). LAB strains could improve rumen DM digestibility by interacting with rumen microorganisms (Wang et al., 2019b). Compared with the GFJ treatment, inoculation with YC1, PC3, and BP17 enhanced (*P* < 0.05) the IVDMD of PM silage. This is because PM silage inoculated with YC1, PC3, and BP17 had a lower pH, which inhibited the growth of other microorganisms, thus reducing dry matter loss during silage fermentation. However, the *in vitro* DM digestibility of PM silage with GFJ was lower than that of PM silage without LAB. Similar result from Weinberg et al. (2007), who reported that the *in vitro* DM digestibility of wheat silage with LAB was lower than that of wheat silage without LAB. Based on these results, LAB isolated from different sources could maintain the nutritional value of PM silage.

**TABLE 3:** The chemical compositions of fresh and ensiled paper mulberry.

Samples	DM %FM	CP	WSC	NDF	ADF	IVDMD
		
		% DM				
Fresh						
	22.12	23.58	7.58	30.39	18.3	–
Silage						
YC1	33.88	19.01b	2.25b	27.09a	14.88a	89.93a
PC3	32.57	20.33a	2.62b	22.22b	10.64b	90.02a
BP17	33.01	19.78a	1.81c	19.51b	10.08b	92.86a
CK	33.22	18.65c	3.11a	22.68b	11.23b	89.22a
GFJ	32.69	19.06b	3.01a	27.91a	15.91a	85.18b
SEM	0.96	0.71	0.14	1.95	0.73	3.00
*P*-value	0.131	0.003	0.001	0.001	0.004	0.007

^a–c^Means in the same column with different letters differ significantly from each other (P < 0.05).

DM, dry matter; FM, fresh matter; CP, crude protein; WSC, water-soluble carbohydrate; NDF, neutral detergent fiber; ADF, acid detergent fiber; IVDMD, in vitro dry matter digestibility; YC1, Lactiplantibacillus plantarum; PC3, Levilactobacillus brevis; BP17, Lactiplantibacillus plantarum; CK, control without additives, applied at 5 mL kg^–1^ FM 0.9% physiological saline; GFJ, Gaofuji, a commercial inoculant containing Lactiplantibacillus plantarum and Lentilactobacillus buchneri; SEM, standard error of means.

### Fermentation quality of ensiled paper mulberry

The fermentation quality of PM silage is shown in Table 4. The LAB treatments had significant effects (*P* < 0.05) on the pH value and NH_3_-N, LA, and AA levels of PM silage. Compared with the CK and GFJ treatments, the BP17 and PC3 treatments had lower (*P* < 0.05) pH values and NH_3_-N contents. This is likely because the LAB could convert WSC into LA, resulting in a decrease in pH and an increase in the LA content (You et al., 2021). However, since LAB activity plays an important role in LA accumulation and pH reduction in the early stages of ensiling (Davies et al., 1998), LAB from different sources have different effects on the silage quality of PM. In addition, the BP17 treatment had the lowest pH (4.89) and the highest LA content (10.94% DM). This result was attributed to the high count (10^8.63^ cfu/g FM) of LAB contained in the BP17 treatment. However, although the PC3 treatment contained a large number of LAB (10^8.92^ cfu/g FM), the LA level of silage was lower than that of the BP17 and YC1 treatments. This is because PC3 is a heterofermentative LAB (*Levilactobacillus brevis*) that can convert LA to AA (Danner et al., 2003), so the PC3-treated silage had the highest (*P* < 0.05) AA content (1.83% DM) (Table 4). The NH_3_-N content of silage is an indication of the degree of proteolysis (Wang et al., 2018b). Previous studies have suggested that well-preserved silage should contain NH_3_-N < 10% TN (Umaña et al., 1991). However, the NH_3_-N content (6.11% TN-9.12% TN) of all silages in our study was below the recommended levels. In addition, the NH_3_-N content in all LAB-treated silages was lower (*P* < 0.05) than that in the CK silage. This result confirmed that LAB reduced the pH value of the silage environment, thereby inhibiting the development and proteolytic activity of other microorganisms (such as clostridia) (Heinritz et al., 2012). PA and BA are undesirable because they waste metabolic energy in their creation (Dong et al., 2020). No PA or BA was detected in any PM silage in our study, which is consistent with the results of Cheng et al. (2021a).

**TABLE 4:** The fermentation quality of paper mulberry silages.

Samples	pH	NH_3_-N	LA	AA
		
		(%TN)	(%DM)	(%DM)
YC1	4.98bc	6.72b	6.40b	0.82bc
PC3	4.90c	6.11c	4.51c	1.83a
BP17	4.89c	6.15c	10.94a	1.09b
CK	5.35ab	9.12a	4.47c	0.62c
GFJ	5.42a	7.19b	4.57c	0.77bc
SEM	0.08	1.17	0.68	0.12
*P*-value	0.021	<0.001	<0.001	<0.001

^a–c^Means in the same column with different letters differ significantly from each other (P < 0.05).

NH_3_-N, ammonia nitrogen; TN, total nitrogen; LA, lactic acid; AA, acetic acid; DM, dry matter; YC1, Lactiplantibacillus plantarum; PC3, Levilactobacillus brevis; BP17, Lactiplantibacillus plantarum; CK, control without additives, applied at 5 mL kg^–1^ FM 0.9% physiological saline; GFJ, Gaofuji, a commercial inoculant containing Lactiplantibacillus plantarum and Lentilactobacillus buchneri; SEM, standard error of means. No propionic acid or butyric acid was detected in any paper mulberry silage.

### Alpha diversities of bacteria and fungi in fresh and ensiled paper mulberry

The sequencing information and bacterial diversity analysis of ensiled PM are shown in Table 5. The Good’s coverage of all samples was more than 0.99, suggesting that the sequencing results could reveal the bacterial diversity of PM silage. Inoculation with LAB had a significant (*P* < 0.05) effect on the number of OTUs and on the Shannon, Ace and Chao1 indexes of PM silage. During ensiling, a decrease in observed species was found in YC1-, PC3-, and BP17-treated silages compared with the CK and GFJ-treated silages (*P* < 0.05). This is likely because many microorganisms are replaced by anaerobic LAB during ensiling, and the LA produced by LAB also inhibits harmful microorganisms. The Shannon index of the PC3-treated silage was decreased compared with that of the CK and GFJ-treated silages (*P* < 0.05). Similar results were found by Wang et al. (2021a), who reported that the Shannon index decreased after inoculating PM silage with *Lentilactobacillus buchneri* in PM silage. However, the Shannon index of the BP17-treated silage was higher (*P* < 0.05) than that of the CK and GFJ-treated silages. This was in accordance with the results from Wang et al. (2021a), who reported that the Shannon index increased after inoculating PM silage with *L. plantarum*. The Ace and Chao1 indexes of the YC1-, PC3-, and BP17-treated silages were lower than those of the CK and GFJ-treated silages (*P* < 0.05). These results agree with the results of Du et al. (2021), who found that the Chao and Ace indexes of PM silage increased after inoculation with LAB compared with the CK. These results suggest that our screened LAB can quickly reduce the pH of PM silage and thus inhibit harmful microorganisms and reduce the alpha diversity of bacteria (Li et al., 2021).

**TABLE 5:** The alpha-diversity of bacterial community of fresh and ensiled paper mulberry.

Samples	Observed species	Shannon	Chao1	Ace	Coverage
Fresh					
	158a	3.98a	165.29a	166.68a	0.9997
Silage					
YC1	100d	3.23b	105.63d	105.47e	0.9998
PC3	98d	2.93c	101.88d	105.04e	0.9998
BP17	111c	3.81a	124.15c	123.53d	0.9997
CK	130b	3.43b	135.25b	138.37c	0.9998
GFJ	142b	3.57b	165.00a	157.81b	0.9996
SEM	24.22	0.38	27.81	26.10	0.00
*P*-value	0.035	0.026	<0.001	<0.001	–

^a–e^Means in the same column with different letters differ significantly from each other (P < 0.05).

YC1, Lactiplantibacillus plantarum; PC3, Levilactobacillus brevis; BP17, Lactiplantibacillus plantarum; CK, control without additives, applied at 5 mL kg^–1^ FM 0.9% physiological saline; GFJ, Gaofuji, a commercial inoculant containing Lactiplantibacillus plantarum and Lentilactobacillus buchneri; SEM, standard error of means; “–,” default.

The sequencing information and fungal diversity of PM silage are shown in Table 6. The majority of research on fungi has focused on toxin-producing fungi (Duniere et al., 2017), but few studies have investigated the epiphytic fungal community in silage. In our study, the Good’s coverage of all samples was more than 0.99, indicating that the sequencing depth was sufficient for revealing the complete fungal diversity. During ensiling, a decrease in the OTUs, Ace and Chao1 indexes was found in LAB silages compared with the CK silage (*P* < 0.05). A consistent result was reported by Keshri et al. (2018), who found that the alpha index of fungi in LAB-treated corn silage was lower than that in CK silage. Therefore, LAB reduces the diversity and richness of the fungal community in PM silage.

**TABLE 6:** The alpha-diversity of fungal community of fresh and ensiled paper mulberry.

Samples	Observed species	Shannon	Chao1	Ace	Coverage
Fresh					
	240c	2.78b	257.53c	263.59c	0.9992
Silage					
YC1	276b	5.47a	287.54b	291.02b	0.9995
PC3	233c	5.20a	249.11c	258.93c	0.9993
BP17	279b	5.15a	302.08b	292.28b	0.9995
CK	316a	5.59a	380.69a	360.49a	0.9990
GFJ	285b	4.75a	308.64b	310.31b	0.9991
SEM	30.70	1.04	47.12	36.95	0.00
*P*-value	0.044	0.035	0.038	0.021	–

^a–c^Means in the same column with different letters differ significantly from each other (P < 0.05).

YC1, Lactiplantibacillus plantarum; PC3, Levilactobacillus brevis; BP17, Lactiplantibacillus plantarum; CK, control without additives, applied at 5 mL kg^–1^ FM 0.9% physiological saline; GFJ, Gaofuji, a commercial inoculant containing Lactiplantibacillus plantarum and Lentilactobacillus buchneri; SEM, standard error of means; “–,” default.

### Bacterial and fungal communities of fresh and ensiled paper mulberry

**FIGURE 1:**
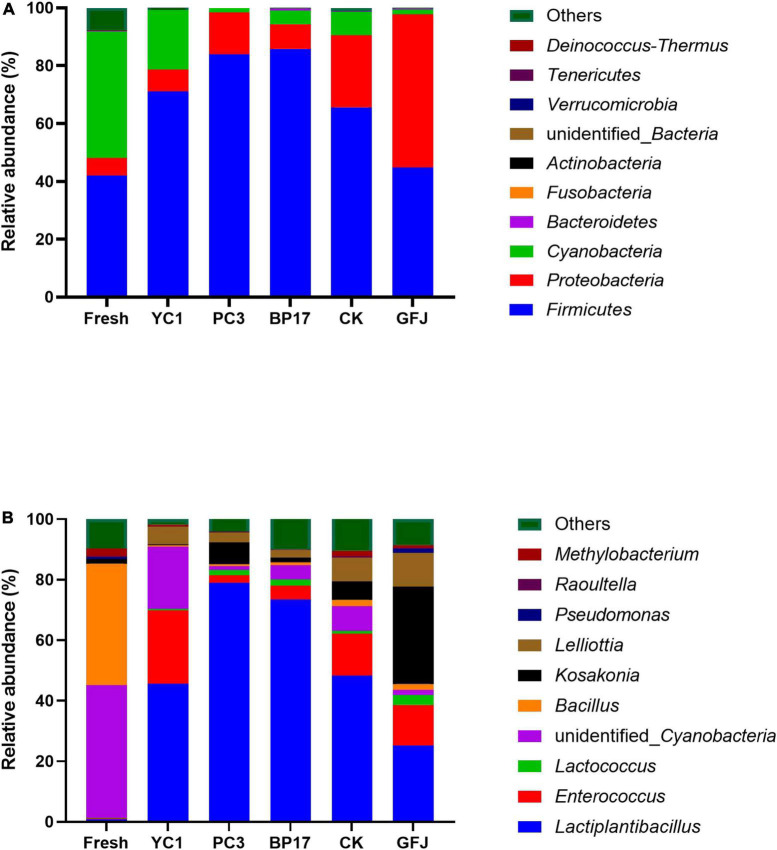
The relative abundance of bacteria at the phylum **(A)** and genus **(B)** levels in fresh paper mulberry and silages inoculated with saline (CK), with a commercial inoculant (GFJ, a combination of *Lactiplantibacillus plantarum* and *Lentilactobacillus buchneri*, 1.0 × 10^6^ cfu/g of FM) or with one of the three selected strains (YC1, *Lactiplantibacillus plantarum*; PC3, *Levilactobacillus brevis*; BP17, *Lactiplantibacillus plantarum*) at 1.0 × 10^6^ cfu/g of FM.

The relative abundance of bacterial communities in the PM silages is shown in Figure 1. The main phyla detected in fresh PM were *Firmicutes*, *Cyanobacteria*, and *Proteobacteria* (Figure 1A). This is in accordance with the results from He et al. (2021), who reported that these bacteria were present in fresh and ensiled PM. *Firmicutes* and *Proteobacteria* were the main phyla detected in the PM silages. Compared to the fresh PM, the PM silages showed decreased relative abundances of *Cyanobacteria* and increased relative abundances of *Firmicutes*. Similar results were obtained by He et al. (2021), who reported that the relative abundance of *Cyanobacteria* in PM silages was lower and the relative abundance of *Firmicutes* was higher than those in fresh PM. Compared with the CK silage, the YC1-, PC3-, and BP17-treated silages had lower relative abundances of *Cyanobacteria* and *Proteobacteria* and a higher relative abundance of *Firmicutes*. The results of our study agree with those of Liu et al. (2019), who found that LAB-treated silage exhibited greater *Firmicutes* abundance and lower *Proteobacteria* and *Cyanobacteria* abundances than the CK silage. However, the relative abundance of *Cyanobacteria* was decreased and the relative abundance of *Proteobacteria* was increased in the GFJ-treated silage compared with other silages. This result indicated that GFJ did not improve the fermentation quality of PM silage. Similar results were obtained by Wang et al. (2021a), who reported that inoculation with LAB derived from *Leymus chinensis* silage had no discernible effect on the fermentation quality of PM silage. As illustrated in Figure 1B, *Cyanobacteria* and *Bacillus* were the predominant genera in fresh PM; these genera are not conducive to silage fermentation, and LAB treatments need to be added to improve the fermentation quality of PM silage. In the present study, *Lactiplantibacillus* and *Enterococcus* were the predominant genera in the PM silages. This was consistent with the findings of Cheng et al. (2021b), who showed that the predominant bacteria in the PM silage inoculated with LAB were *Enterococcus* and *Lactiplantibacillus*. A variety of beneficial LAB, including *Lactiplantibacillus*, *Weissella*, *Lactococcus*, *Leuconostoc*, and *Streptococcus*, are essential for enhancing LA production and reducing pH (Dong et al., 2020). Compared with the CK and GFJ-treated silages, the PC3- and BP17-treated silages had increased relative abundances of *Lactiplantibacillus* and decreased relative abundances of *Enterococcus*. Since *Lactiplantibacillus* is strongly acid tolerant, it would remain unaffected at low pH values, while less acid-tolerant microorganisms such as *Enterococcus* would be inhibited (Li et al., 2021). In the present study, the relative abundance of *Kosakonia* in the GFJ-treated silage was higher than that in the other silages. Studies have found that *Kosakonia*, a bacterium similar to *Enterobacter*, can produce NH_3_-N, leading to silage spoilage (Kosako et al., 1996; Duniere et al., 2013). Compared with the CK and GFJ-treated silages, the PC3- and BP17-treated silages showed decreased relative abundances of *Lelliottia*. *Lelliottia* has been rarely reported to be distributed in silage. *Lelliottia*, an *Enterobacteriaceae* member (Yuk et al., 2018), can create NH_3_-N, resulting in poor silage quality.

**FIGURE 2:**
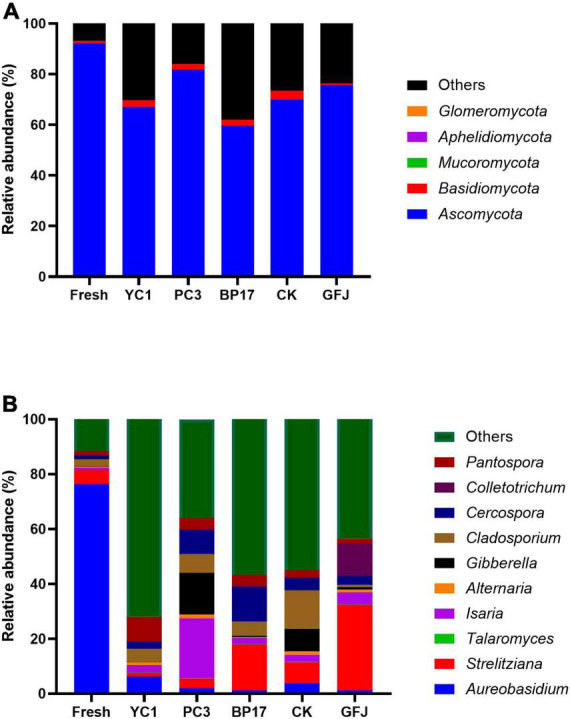
The relative abundance of fungi at the phylum **(A)** and genus **(B)** levels in fresh paper mulberry and silages treated inoculated with saline (CK), with a commercial inoculant (GFJ, a combination of *Lactiplantibacillus plantarum* and *Lentilactobacillus buchneri*, 1.0 × 10^6^ cfu/g of FM) or with one of the three selected strains (YC1, *Lactiplantibacillus plantarum*; PC3, *Levilactobacillus brevis*; BP17, *Lactiplantibacillus plantarum*) at 1.0 × 10^6^ cfu/g of FM.

The fungal composition of PM silages is shown in Figure 2. Since well-fermented silages do not contain toxin-producing fungi, researchers rarely consider the fungal community in silage (Duniere et al., 2017). The main phyla detected in the fresh PM were *Ascomycota* and *Basidiomycota* (Figure 2A). A similar result was found in Zhang et al. (2019b), which reported that *Ascomycota* were highly abundant, followed by *Basidiomycota*, in silages. Compared to the PM silage, the fresh PM had a higher relative abundance of *Ascomycota* and a lower relative abundance of *Basidiomycota*. However, an opposite result was obtained by Bai et al. (2020), who found that *Ascomycota* abundance increased and *Basidiomycota* abundance decreased in alfalfa silage compared with fresh material. Hence, we attribute that LAB have different effects on the fungal communities of different forage species. The dominant fungal genus in fresh PM was *Aureobasidium* (Figure 2B), which has been reported to be the predominant genus on the skin of grapes (Gao et al., 2019). However, inconsistent results were reported by Chen et al. (2020b), who reported that the dominant fungal genera in fresh PM were *Mortierella* and *Hannaella*. Numerous factors, such as forage types and environmental factors, result in differences in the fungal community composition (Li et al., 2021). The dominant fungal genus in the PM silages was unclassified fungi (Figure 2B), which was similar to the results of Li et al. (2021), who found that unclassified fungi were the most abundant microorganisms in additive-treated timothy silage. Compared with the CK and GFJ-treated silages, the PC3- and YC1-treated silages had increased relative abundances of *Strelitziana*. However, this is the first study to report that *Strelitziana* is distributed in PM silage. Further study is needed to investigate the effect of *Strelitziana* on silage quality. Compared with the CK silage, the BP17- and PC3-treated silages had decreased relative abundances of *Cladosporium*. *Cladosporium*, which produces mycotoxins, is a mold commonly found in plants (Tabuc et al., 2011). The reduction in *Cladosporium* indicated that inoculation with BP17 and PC3 improved the quality of silage. The relative abundance of *Gibberella* was decreased in the BP17-treated silage compared with the CK silage, while it was decreased in the PC3-treated silage. *Gibberella* can produce mycotoxins such as zearalenone, a fungus commonly found in corn (Anderson et al., 2017). Thus, inoculation of PM silage with BP17 could inhibit mycotoxin. However, inoculation with PC3 showed a limited effect on the activity of *Gibberella*. In addition, the highest relative abundance of *Isaria* was observed in the PC3-treated silage. Several studies have shown that *Isaria* could be used for the biotransformation of flavonoids, glycosides, steroids, and other compounds (Dymarska et al., 2018; Kozłowska et al., 2018). There are few reports on *Isaria* in silage; therefore, additional research is needed to investigate the role of *Isaria* in silage.

These results suggested that inoculation with BP17 and PC3 increased the relative abundance of *Lactiplantibacillus* and decreased the relative abundance of *Lelliottia* and *Cladosporium*, resulting in improved PM silage quality.

## Conclusion

This study evaluated the effect of LAB strains from various sources on the silage quality and the PM microbial community. Among the evaluated LAB strains, BP17 isolated from the fresh PM leaves and PC3 isolated from pickle could significantly improve the silage quality of PM, mainly in terms of high CP and LA contents and low pH and NH_3_-N contents. Inoculation with BP17 and PC3 increased the relative abundance of *Lactiplantibacillus* and decreased the relative abundances of *Lelliottia* and *Cladosporium*, thereby improving the silage quality of PM. This study showed that BP17 and PC3 could be used as silage additives.

## Data availability statement

The original contributions presented in this study are included in the article, further inquiries can be directed to the corresponding author.

## Authors contributions

PL and CC designed the experiments and revised the manuscript. ML, XF, YC, HS, YX, and YZ performed the experiments. QC and ML wrote the manuscript. QC carried out the data analysis. All authors reviewed and considered the manuscript.

## Footnotes

1
https://magic.novogene.com
